# Oral Supplementation with *Prunus domestica* L. Extract Restores Recognition Memory Impairment Caused by D-Galactose in Rats

**DOI:** 10.3390/nu17233804

**Published:** 2025-12-04

**Authors:** Anusara Aranarochana, Puncharatsm Pannin, Papatchaya Sintow, Apiwat Sirichoat, Nataya Sritawan, Wanassanan Pannangrong, Rawiwan Charoensup, Wuttichai Jaidee, Piti Ungarreevittaya, Peter Wigmore, Jariya Umka Welbat

**Affiliations:** 1 Department of Anatomy, Faculty of Medicine, Khon Kaen University, Khon Kaen 40002, Thailand; 2 Neurogenesis Research Group, Khon Kaen University, Khon Kaen 40002, Thailand; 3 Medicinal Plant Innovation Center of Mae Fah Luang University, Mae Fah Luang University, Chiang Rai 57100, Thailand; 4 School of Integrative Medicine, Mae Fah Luang University, Chiang Rai 57100, Thailand; 5 Department of Pathology, Faculty of Medicine, Khon Kaen University, Khon Kaen 40002, Thailand; 6 School of Life Sciences, Medical School, Queen’s Medical Centre, University of Nottingham, Nottingham NG7 2RD, UK

**Keywords:** *Prunus domestica* L., phytochemical contents, antioxidant activity, D-galactose, memory impairment, hippocampal neurogenesis

## Abstract

**Background/Objectives:** Aging-related cognitive decline, linked to oxidative stress and impaired hippocampal neurogenesis, is a major contributor to neurodegenerative disorders. In rodents, this condition can be modeled by D-galactose (D-gal) administration, which induces oxidative stress and recognition memory deficits. *Prunus domestica* L. (PD), rich in phenolic and flavonoid compounds with antioxidant properties, may counteract such impairments. This study evaluated the effects of PD extract on D-gal-induced memory decline by analyzing its phytochemical content, antioxidant activity, and neuroprotective potential. **Methods:** Phytochemicals were quantified by colorimetric and pH differential methods, and antioxidant capacity was determined using DPPH and FRAP assays. Male Sprague Dawley rats (12 weeks; n = 12/group) were assigned to 8 groups: vehicle, D-gal, PD (75, 100, or 150 mg/kg), and D-gal + PD (same respective doses). D-gal (50 mg/kg, i.p.) and/or PD were administered by oral gavage daily for 8 weeks. Recognition memory was assessed by the novel object recognition (NOR) test. Hippocampal tissues were processed for immunofluorescence staining of the proliferation marker Ki-67 and superoxide dismutase (SOD) activity using the cytochrome C reduction method. **Results:** PD extract contained abundant phenolics, tannins, flavonoids, and anthocyanins, and exhibited notable antioxidant activity. D-gal impaired recognition memory, reduced hippocampal cell proliferation, and decreased SOD activity. Co-treatment with PD improved memory performance, enhanced hippocampal neurogenesis, and restored antioxidant enzyme activity. **Conclusions:** PD extract may protect against D-gal-induced age-related cognitive decline through antioxidant effects and support of hippocampal neurogenesis.

## 1. Introduction

High consumption of fruits and vegetables is known to promote health, partly due to their content of phenolic compounds. These compounds, synthesized by plants as secondary metabolites, are widely present in various food sources. Previous studies have strongly indicated that phenolic compounds confer protection against major diseases, primarily due to their antioxidant properties [1,2]. Flavonoids, anthocyanins, and tannins—natural phytochemicals—are phenolic compounds known to defend against cellular oxidative stress. Specifically, flavonoids have been shown to mitigate symptoms of neurodegenerative diseases due to their inherent antioxidant properties [3]. Numerous natural flavonoids are found in *Prunus domestica* L. (PD), a species of plum. These fruits are rich in natural phenolic phytochemicals, which potentially serve as powerful antioxidants when consumed as part of a regular diet [4,5]. Historically, plums have been recognized for their diverse pharmacological activities, including reducing the effects of aging and preventing Alzheimer’s disease [6,7]. The implementation of PD has shown a positive effect in countering age-related declines in cognitive behavior. This evidence indicates that PD supplementation mitigates age-related impairments in cognitive performance, indicating its potential to improve memory and learning in models of aging rats [8]. Polyphenolic compounds contained in PD, particularly chlorogenic acid, quercetin, and rutin, have been reported to exhibit potent antioxidant and neuroprotective properties [9]. These bioactive molecules activate the Keap1/Nrf2/ARE signaling cascade, leading to the upregulation of endogenous antioxidant enzymes such as SOD, CAT, and GPx, and the attenuation of oxidative damage. This effect has also been demonstrated in related Prunus species such as *P. spinosa* [10]. Beyond its antioxidant function, Nrf2 regulates neural stem cell proliferation and differentiation within the hippocampal neurogenic niche, and its age-related decline is therefore associated with reduced neurogenesis and cognitive performance [11]. In parallel, chlorogenic acid and quercetin modulate the cAMP/PKA–CREB–BDNF pathway, enhancing BDNF expression, neuronal survival, and hippocampal plasticity [12,13]. Collectively, activation of the Nrf2 and BDNF pathways provides a mechanistic basis for the antioxidant and neurogenic potential of PD extract. Although PD has shown cognitive-enhancing and antioxidant effects in scopolamine-induced models [14], no systematic studies have yet investigated its efficacy in D-galactose–induced brain aging or oxidative cognitive impairment. Brain aging correlates with increased reactive oxygen species (ROS) levels, which induce oxidative stress. This stress causes neuronal damage, degeneration, and subsequent loss. This degeneration compromises neuroplasticity and diminishes neurogenesis in the subgranular zone (SGZ) of the hippocampal dentate gyrus (DG)—a pivotal neurogenic region responsible for producing neurons that contribute to learning and memory processes like spatial working memory [15,16]. The age-induced decline in neurogenesis is tied to the degeneration of neural stem cells in the hippocampal zone. This decline arises from cellular aging due to an accumulation of free radicals, causing oxidative stress on neurons, ultimately leading to neuronal death and a subsequent decline in hippocampal-dependent memory [17]. Additionally, aging in brain demonstrates a depletion in BDNF, which results to hippocampal neurogenesis impairment [18,19]. It is therefore important to explore the potential of *Prunus domestica* L. (PD) supplementation beyond its antioxidant capacity, as its neuroprotective effects during aging remain obscure. Therefore, this study aimed to examine the phytochemical composition and antioxidant potency of freshly harvested PD fruits collected from northern Thailand, extracted using ethanol, and to evaluate their neuroprotective effects against hippocampal neurogenesis and memory impairments in D-galactose–induced aging in rats.

## 2. Materials and Methods

### 2.1. Plant Extraction

Fresh *Prunus domestica* L. (PD) fruits were collected in late February 2021 from the Mae Chan District in Chiang Rai, Thailand. The collection was authenticated by Assoc. Prof. Dr. Rawiwan Charoensup. The PD specimen has been assigned a voucher number (MPIC0174) and deposited at the Queen Sirikit Botanic Garden Herbarium (NO. 105711). Mature, undamaged fruits were macerated in 95% ethanol and then filtered through paper to obtain the supernatant. This supernatant was then subjected to rotary evaporation to yield a powder, and the percentage yield of the PD crude extract was calculated. The PD crude extract was stored at −20 °C until further analysis.

### 2.2. UHPLC-QTOF-MS Analysis

The chemical constituents of the PD crude extract were analyzed using ultra-high-performance liquid chromatography coupled with quadrupole time-of-flight mass spectrometry (UHPLC-QTOF-MS). The dried PD extract (1.0 mg) was dissolved in acetonitrile and filtered through a 0.22 μm membrane to prepare a stock solution, which was further diluted to a final concentration of 200 μg/mL. The dried extract was stored at −20 °C until analysis. LC-MS/MS analysis was carried out at the Medicinal Plant Innovation Center, Mae Fah Luang University (Thailand), using an Agilent 1290 Infinity II UHPLC system coupled to an Agilent 6545B Q-TOF mass spectrometer (Agilent Technologies, Santa Clara, CA, USA). Separation was performed on an Agilent ZORBAX Eclipse Plus C18 column (2.1 × 50 mm, 1.8 μm, Agilent Technologies, Santa Clara, CA, USA) maintained at 30 °C, with a mobile phase consisting of 0.1% formic acid in water (A) and 0.1% formic acid in acetonitrile (B). The gradient elution program was set as follows: 5% B (0–1 min), 5–17% B (1–10 min), 17% B (10–13 min), 17–100% B (13–20 min), 100% B (20–25 min), 100–5% B (25–27 min), and 5% B (27–30 min). The flow rate was 1.0 mL/min, and the injection volume was 1.0 μL.

The mass spectrometer was equipped with a Dual AJS ESI source operated in positive ion mode under the following conditions: capillary voltage, 3500 V; nebulizer pressure, 35 psi; drying gas flow, 10 L/min at 325 °C. The spectra were recorded over an m/z range of 100–1000 at a scan rate of 2 spectra/s. Data acquisition and processing were performed using Agilent MassHunter Workstation software (version B.08.00) and MassHunter Profinder (version 10.0). Compound identification was achieved through elemental composition prediction, MS/MS fragment ion matching, and comparison with spectral libraries using a mass error tolerance of less than 5 ppm. The relative abundance of each metabolite class was estimated from the percentage of its peak area in the total ion chromatogram (TIC) obtained in both positive and negative ionization modes. The LC–MS/MS analysis was performed in triplicate (n = 3).

### 2.3. Cytotoxic Activity Assays

We utilized the murine macrophage RAW264.7 cell line to evaluate the cytotoxic effects of the PD extract. The RAW 264.7 cell line was obtained from the American Type Culture Collection (ATCC, Manassas, VA, USA, Cat. No. TIB-71). The reference genome of Mus musculus is available under accession number GCF_000001635.27. This selection was informed by the cell line’s established utility in analogous assays and its ability to produce consistent and reproducible results. The choice to employ RAW264.7 cells aligns with recognized methodologies in studies involving plant extracts. This decision offers a dependable model for preliminary cytotoxicity screening [20,21]. The cytotoxic effect of the PD extract was evaluated using an MTT assay, as described in van Meerloo et al. (2011) [22]. The RAW264.7 cell line was exposed to various concentrations of the PD extract (ranging from 3.91 to 500 µg/mL) in a 96-well plate for 24 h. Subsequently, MTT solution at a concentration of 0.5 mg/mL was added to the RAW264.7 cell line and incubated at 37 °C to assess cell viability. Viable cells can reduce tetrazolium salts to insoluble, purple-colored formazan crystals. The intensity of this colored product, measured spectrophotometrically with an absorbance maximum near 570 nm, is directly proportional to the number of viable cells after exposure to the PD extract. All experiments were performed with three biological replicates, each measured in triplicate (technical replicates). The data were expressed as mean ± SD.

### 2.4. Total Phenolic Compounds

The Folin–Ciocalteu assay [23] was performed to determine the total phenolic compounds in the PD crude extract. The extract was prepared in ethanol and serially diluted, using gallic acid as the standard. After incubation with Folin–Ciocalteu reagent and sodium carbonate in the dark, absorbance was measured at 765 nm using a UV–Vis spectrophotometer (Evolution™ 300, Thermo Fisher Scientific, Darmstadt, Germany). The phenolic content of the PD crude extract was quantified and expressed as mg gallic acid equivalent (GAE) per g of extract.

### 2.5. Total Tannin Content

The total tannin content of the PD crude extract was determined using the Folin–Ciocalteu phenol reagent method, adapted from Son et al. (2013) [24]. Serial dilutions of the extract in ethanol were analyzed in parallel with tannic acid standards. After reaction with Folin–Ciocalteu reagent and sodium carbonate, samples were incubated in the dark for 2 h, and absorbance was measured at 725 nm. The tannin content was quantified and expressed as mg tannic acid equivalent (TAE) per g of extract.

### 2.6. Total Flavonoid Content

The total flavonoid content of the PD crude extract was determined using the aluminum chloride colorimetric method, modified from a previous study [25]. Serial dilutions of the extract in ethanol were analyzed in parallel with quercetin standards, and the mixtures were incubated with aluminum chloride and sodium acetate solutions in the dark for 15 min. Absorbance was measured at 430 nm, and the flavonoid content was expressed as mg quercetin equivalent (QE) per g of extract.

### 2.7. Total Anthocyanin Content

The total anthocyanin content of the PD crude extract was determined using the pH differential method [26]. The extract was diluted with buffers at pH 1.0 (0.025 M hydrochloric acid–potassium chloride) and pH 4.5 (0.4 M sodium acetate) at a ratio of 1:15. After 30 min of incubation, absorbance was measured at 510 and 700 nm, and the anthocyanin content was quantified as cyanidin-3-glucoside equivalents using the following equation:

Monomeric anthocyanin pigment (mg/L) = (A × MW × DF × 1000)/ε

where

- MW represents the molecular weight of cyanidin-3-glucoside (449.2)
- DF denotes the dilution factor (15)
- ε is the molar absorptivity of cyanidin-3-glucoside (26,900)
- A is calculated as: A = (A_510_ − A_700_)_pH 1.0_ − (A_510_ − A_700_)_pH 4.5_

### 2.8. Determination of 2,2-Diphenyl-1-picryl-hydrazyl (DPPH) Radical Scavenging Activity

The free radical scavenging activity of the PD crude extract was evaluated spectrophotometrically at 517 nm using the DPPH method, as adapted from Brand-Williams et al. (1995) [27]. L-ascorbic acid was used both as the calibration standard for expressing antioxidant activity and as the positive control for comparison of radical scavenging efficiency. Serial ethanol dilutions of the extract and L-ascorbic acid were each combined with DPPH solution and kept in the dark for 30 min. The IC_50_ values, representing the concentration required to scavenge 50% of DPPH free radicals, were calculated for both the extract and L-ascorbic acid using the following equation: DPPH radical scavenging (%) = [(Abs_control_ − Abs_sample_)/Abs_control_] × 100, where Abs_control_ is the absorbance of the control and Abs_sample_ is the absorbance of the sample.

### 2.9. Determination of Ferric Reducing Antioxidant Power (FRAP)

The ferric-reducing power of the PD crude extract was assessed based on the principle that an antioxidant converts the ferric–TPTZ complex into its ferrous form, which produces a blue complex under acidic conditions. The extract was serially diluted in ethanol and compared with L-ascorbic acid standards. Subsequently, the diluted extract was mixed with freshly prepared FRAP reagent. After a 10 min incubation period at 37 °C, absorbance was recorded at 593 nm. The FRAP value was derived from the linear standard curve of L-ascorbic acid and expressed as µM L-ascorbic acid equivalent per mg of extract (µM L-ascorbic acid/mg extract) [28].

### 2.10. Animals and Drug Administration

A priori sample size estimation based on pilot data from Zhao et al. (2019) [29] for a two-sample, two-sided *t*-test (α = 0.01, power = 0.80; Δ = 23.22, pooled σ = 15.16; Cohen’s d = 1.53) indicated that a minimum of 10 rats per group was required. Therefore, 12 rats per group were used to ensure adequate statistical power and to compensate for potential attrition [30] (see Supplementary File S1: Sample Size and Power Calculation).

Ninety-six adult male Sprague–Dawley (SD) rats, aged 8 weeks and weighing 280–300 g, were obtained from Nomura Siam International Co., Ltd., Bangkok, Thailand. To minimize baseline variation, rats were initially housed in standard cages by the Animal Facility staff. All experimental procedures strictly adhered to ethical guidelines and received approval from the Khon Kaen University Ethics Committee in Animal Research (protocol code: AEKKU 49/64; approval date: 20 May 2021). The investigator then reorganized the animals into treatment groups based on body weight to ensure comparable mean weights across all groups before treatment commenced. The Animal Facility staff handled daily animal care and group allocation, who was not involved in routine handling, performed all experimental procedures and data collection. This strict separation of duties helped maintain consistency and minimize potential observer bias. The rats were housed in groups of four per standard plastic cage with wood shaving bedding in a well-ventilated room maintained at 23–25 °C with a 12:12 h light/dark cycle (lights on at 6:00 a.m.). All rats received a standard balanced diet and had free access to food and tap water ad libitum. The standard rodent diet was a commercially available, pasteurized formulation produced by Perfect Companion Group Co., Ltd. (Sathorn District, Bangkok, Thailand) and was designed to meet the basic nutritional requirements of laboratory rodents. The diet contained not less than 18% protein and 3% fat, with a maximum of 24% fiber, 10% moisture, and 10% ash. It was also fortified with essential vitamins, including a minimum of 9000 IU/kg of vitamin A, 1800 IU/kg of vitamin D_3_, 80 IU/kg of vitamin E, and 800 mg/kg of vitamin C. The composition and production process adhered to established laboratory animal nutrition guidelines to ensure animal health and experimental consistency. Before the experimental procedures, all rats underwent a 4-week acclimatization period without drug administration. Thereafter, the rats were randomly assigned to eight groups for an 8-week drug administration protocol. Group 1 (vehicle group) received saline via intraperitoneal (i.p.) injection and distilled water orally, whereas Group 2 (aging model group) was administered D-galactose at 50 mg/kg to induce aging. PD crude extracts were given orally to rats in dosages of 75, 100, and 150 mg/kg for Groups 3, 4, and 5, respectively. Groups 6, 7, and 8 consisted of rats treated with D-galactose at the same concentration as Group 2 via i.p. injection, along with oral administration of PD crude extracts at dosages of 75, 100, and 150 mg/kg, respectively. The degree of stress induced by different administration methods, such as oral gavage and intraperitoneal injection, has been shown to vary depending on several factors, including animal age, injection frequency, handling techniques, and habituation. Some studies, including those involving aging rat models, have reported minimal or attenuated stress responses under well-controlled conditions [31,32]. Based on these findings and the careful handling protocols implemented in our study, it is reasonable to assume that any stress experienced by the animals was minimal, although this cannot be fully ruled out.

### 2.11. Behavioral Test

After 8 weeks of drug administration, all rats underwent an assessment of their recognition memory using the novel object recognition (NOR) test as described in Aranarochana et al. (2019) [33] with minor modifications. The equipment consisted of a square open-field arena (50 × 50 × 50 cm), two non-toxic plastic objects of similar size and weight (≈8 cm in diameter × 24 cm in height), and a novel object of a different shape (≈24.5 × 17.5 × 21 cm). The arena was illuminated by controlled overhead lighting at approximately 10 lux. Data acquisition was facilitated by an overhead digital camera linked to a computer-based tracking system (EthoVision^®^, XT version 12, Noldus, Wageningen, The Netherlands). The rats were permitted 30 min of uninhibited exploration in an open-field arena. After 24 h, they were again allowed to explore the arena for 3 min, this time without any stimuli. During the familiarization phase of the NOR test, rats had 3 min to engage with two identical objects, followed by a 15 min inter-trial interval. In the choice phase, one familiar object (FO) and a novel object (NO) were presented at the same locations within the arena, and the rats were given another 3 min to explore. Active exploration was defined by actions such as sniffing, licking, or having the nose oriented towards the object at a proximity of ≤2 cm. The time spent by rats exploring during both tests was recalibrated to a preference index (PI), denoting the time dedicated to novel exploration during the choice trial as a percentage relative to a 50% chance [34].

### 2.12. Immunohistochemistry for Hippocampal Proliferation Ki-67 Marker

Following behavioral testing, the rats were euthanized via rapid stunning followed by cervical dislocation. One hemisphere of each brain was immediately cryoprotected in 30% sucrose dissolved in phosphate-buffered saline. Subsequently, the cryoprotected hemispheres were immersed in Optimal Cutting Temperature (OCT) compound (Thermo Fisher Scientific, Darmstadt, Germany) and rapidly snap-frozen in isopentane cooled with liquid nitrogen (Sigma-Aldrich, St. Louis, MO, USA). These hemispheres were then maintained at −80 °C for immunohistochemistry. Frozen hemispheres were sectioned serially along the dentate gyrus (DG) length (Bregma point −2.3 to −6.3 mm) in the coronal plane at 20 μm thickness. The sections were affixed to gelatin-coated slides and stored at −20 °C for Ki-67 immunostaining. We employed a systematic random sampling technique to select nine sections per hemisphere from every 15 sections along the DG [35]. Initially, sections were incubated with a monoclonal mouse anti-Ki-67 antibody (NOVOCASTRA, Newcastle, UK; 1:150) for 1 h at room temperature, followed by incubation with Alexa Fluor 488 conjugated rabbit anti-mouse IgG (Invitrogen, Waltham, MA, USA, A11059; 1:250) and subsequently counterstained for 30 s with DAPI (Molecular Probes, Eugene, OR, USA; 1:6000). Cell proliferation evaluation utilized a 40× objective on a fluorescence microscope (Nikon Eclipse Ci-L, Nikon Instruments, Inc., Melville, NY, USA), which was interfaced with a digital camera and NIS-Elements software version 5.42.03 for cell counting. Ki-67 positive cells, identified by the fluorescein isothiocyanate (FITC-green) filter, were counted within three cell body distances in the subgranular zone (SGZ) [36]. The count of Ki-67 positive cells was adjusted by a factor of 15 [31,32].

### 2.13. Determination of Superoxide Dismutase (SOD) Activity

Hippocampal and prefrontal cortex tissues were dissected from one hemisphere of each brain. Tissues were homogenized in deionized water and centrifuged, after which the supernatant was collected for protein quantification and SOD activity measurement. Protein concentration was determined using a NanoDrop™ 2000 spectrophotometer (Thermo Scientific, Wilmington, DE, USA) and expressed in mg/mL. SOD activity was assessed using a modified version of the cytochrome C reduction method by McCord and Fridovich in 1969, in which xanthine-xanthine oxidase generates superoxide radicals that reduce cytochrome C. The reaction mixture (216 mM phosphate buffer, 10.7 mM EDTA, 1.1 mM cytochrome C, 0.108 mM xanthine) was combined with 25 µL of sample and the reaction was initiated by the addition of xanthine oxidase. Enzymatic activity was calculated by recording the absorbance at 540 nm at 0 min and 5 min. This measurement was conducted in endpoint mode rather than continuous kinetic acquisition. SOD activity was then calculated based on the change in absorbance between these two time points, using a standard curve generated with 100 U/mL SOD (Sigma-Aldrich, St. Louis, MO, USA), and expressed as U/mg protein [37].

### 2.14. Statistical Analysis

Experimental results were statistically evaluated using both Student’s *t*-test and two-way analysis of variance (ANOVA). All data processing was executed with GraphPad Prism (Version 9.0; GraphPad Software Inc., San Diego, CA, USA). Outcomes are presented as the mean ± standard error of the mean (SEM), with a *p*-value of <0.05 indicating statistical significance.

## 3. Results

### 3.1. UHPLC-QTOF-MS Analysis of Phytochemical Constituents in Prunus domestica Extracts

The UHPLC-QTOF-MS analysis, as described in the Materials and Methods section, revealed a diverse chemical profile of the PD crude extract, comprising 17 identified compounds belonging to phenolic acids, flavonoids, xanthones, terpenoids, and fatty acids. Based on the relative abundance estimated from the total ion chromatogram, fatty acids were the most represented group (≈38% of total peak area), followed by phenolic compounds (≈22%), xanthones (≈13%), terpenoids (≈9%), and other minor metabolites (≈18%). In the positive ionization mode, major constituents included sucrose, oleacein, palmitic acid, lauric acid, 2-pentadecylfuran, and lithocholic acid, whereas catechin, myrsinone, mangostinone, and garcinone A predominated in the negative mode. The chromatographic profiles obtained in both ionization modes are shown in Figure S1, and the identified compounds are listed in Table S1.

### 3.2. Evaluation of the Cytotoxic Effect of Prunus domestica Extracts

At concentrations ranging from 7.81 to 125 µg/mL, the PD extract demonstrated no cytotoxic effect on the RAW264.7 mouse macrophage-like cell line, with a survival rate exceeding 80%. However, at concentrations of 250–500 µg/mL, the PD extract exhibited mild cytotoxicity to the macrophage cell line. All tested concentrations of PD extract yielded an IC_50_ value greater than 500 μg/mL, as detailed in Table 1.

### 3.3. Quantification of the Total Phenolic Compounds in Prunus domestica Extracts

**Figure 1:**
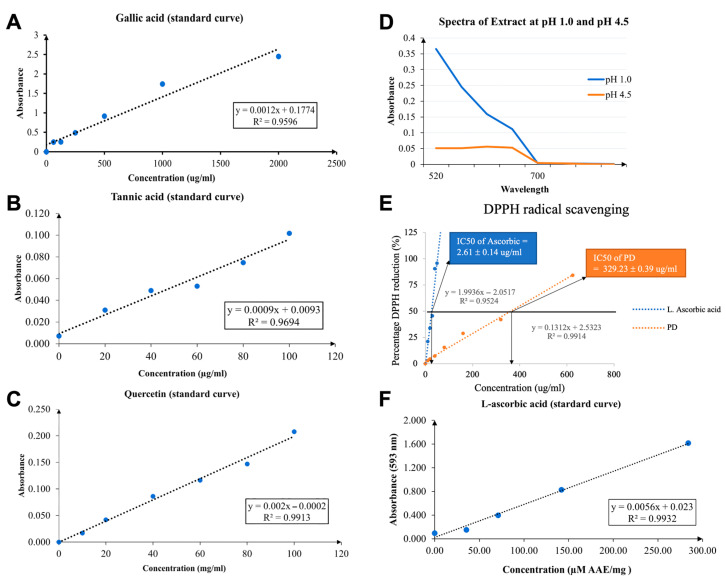
Representative images of the standard curves and spectrum manner for quantifying phytochemical contents and determining antioxidant activities in *Prunus domestica* L. extract (**A**). The gallic acid standard curve (**B**). The tannic acid standard curve (**C**). The quercetin standard curve (**D**). Spectral characteristics of anthocyanin in PD crude extract buffered solutions at pH values 1.0 and 4.5 (**E**). Comparison of DPPH radical scavenging activity (IC50 value) of standard L-ascorbic acid and PD extracts (**F**). The FRAP value (µM AAE/mg extract) of the L-ascorbic acid standard curve. Each data point on the standard curves represents the average of three measurements per sample. DPPH: 2,2-diphenyl-1-picryl-hydrazyl; AAE: ascorbic acid equivalent.

Utilizing the Folin–Ciocalteu reagent assay, the total phenolic compound content of the PD solvent extract was quantified as 19.7 ± 0.02 mg GAE/g of extract. Absorbance values for gallic acid, spanning concentrations from 50 to 2000 μg/mL, were measured at a wavelength of 765 nm. The PD crude extract underwent a two-fold serial dilution with distilled water, beginning with a concentration of 10 mg/mL. These data points were used to create a standard curve, plotting final concentration against absorbance, as shown in Figure 1A.

### 3.4. Quantification of the Total Tannin Content of Prunus domestica Extracts

The Folin–Ciocalteu method was employed in this study to assess the tannin content of the PD extract. Using tannic acid as the reference standard, absorbance was gauged at a wavelength of 725 nm across concentrations ranging from 20 to 100 μg/mL. The PD crude extract was also subjected to a two-fold serial dilution with distilled water, starting from 10 mg/mL. From this, a standard curve was plotted, comparing the final concentration with the absorbance, as illustrated in Figure 1B. The aggregate tannin content in the PD solvent extract was determined to be 34.7 ± 0.06 mg TAE/g of extract.

### 3.5. Quantification of the Total Flavonoid Content of Prunus domestica Extracts

The total flavonoid content of the PD solvent extract was evaluated using the aluminum chloride colorimetric assay. This assay determined the total flavonoid content to be 2.49 ± 0.00 mg QE/g of the extract. Absorbance values for quercetin, spanning concentrations from 10 to 100 mg/mL, were measured, with a two-fold serial dilution performed on the PD crude extract prepared with distilled water. Absorbance values for both quercetin and PD were captured at a wavelength of 430 nm. A standard curve plotting concentration against absorbance was generated to represent these findings, as shown in Figure 1C.

### 3.6. Quantification of the Total Anthocyanin Content of Prunus domestica Extracts

The anthocyanin content in the PD extract was determined by measuring the absorbance changes at two pH levels: 1.0 and 4.5. The PD crude extract underwent dilution using two different buffers: a 0.025 M hydrochloric acid-potassium chloride buffer (pH = 1) and a 0.4 M sodium acetate buffer (pH = 4.5). After the addition of the buffer, absorbance readings were taken at wavelengths of 510 nm and 700 nm, as illustrated in Figure 1D. From the spectral analysis, the derived absorbance was 0.31. The total anthocyanin content in the PD crude extract, expressed in cyanidin-3-glucoside equivalents, was calculated using the following equation: monomeric anthocyanin pigment = (0.31 × 449.2 × 15 × 1000)/26,900, resulting in a value of 77.6 mg/L.

### 3.7. Determination of Antioxidant Capacity of Prunus domestica Extracts

The radical scavenging activity of the PD extract was evaluated using the DPPH assay. Serial concentrations of both L-ascorbic acid and PD extract were tested to calculate their IC50 values, with L-ascorbic acid serving as the positive control. Seven final concentrations of L-ascorbic acid, ranging from 10 to 1000 μg/mL, were utilized. The PD extract underwent serial two-fold dilution, starting with a concentration of 10 mg/mL of the PD crude extract, as illustrated in Figure 1E. A scatterplot was generated, depicting the percent DPPH radical scavenging activity relative to the PD extract concentrations (expressed in μg/mL). The IC50 values (indicating the concentrations of the PD extract that caused a 50% reduction in DPPH radical activity) for L-ascorbic acid and PD extract were calculated at 2.61 ± 0.14 and 329.23 ± 0.39 μg/mL, respectively.

### 3.8. Determination of Ferric Reducing Antioxidant Power (FRAP) of Prunus domestica Extracts

The PD extract’s ferric-reducing ability, indicating its antioxidant capacity, was assessed using the FRAP assay. FRAP values were calculated by referencing the L-ascorbic acid standard curve (Figure 1F). The PD crude extract was serially diluted two-fold to concentrations from 10 to 100 mg/mL. Absorbance readings were taken at 593 nm, and results were expressed as micromolar ascorbic acid equivalents (µM AAE/mg crude extract). The PD solvent extract demonstrated a robust ferric-reducing antioxidant capacity (63.8 µM AAE/mg).

### 3.9. Effects of D-Gal and PD Crude Extract on Locomotor Activities

Locomotor activity was assessed by measuring the total distance moved. Data were analyzed using two-way ANOVA with treatment and dose of PD extract as factors. The analysis revealed a significant interaction between treatment dose and D-gal administration (F(3, 40) = 3.945, *p =* 0.0148). However, neither the main effect of dose (F(3, 40) = 0.9092, *p =* 0.4452) nor the main effect of D-gal (F(1, 40) = 0.2957, *p =* 0.5896) reached statistical significance. Post hoc analysis using Bonferroni’s multiple comparisons test showed no significant differences in total distance moved between vehicle-treated and D-gal-treated groups (*p >* 0.9999). Likewise, treatment with PD extract at all tested doses did not significantly alter locomotor activity in either D-gal-treated or untreated rats (all *p >* 0.7655). These findings suggest that none of the treatments used in this study, including D-gal and PD extract at the tested doses, significantly affected locomotor activity.

### 3.10. Effects of D-Gal and PD Crude Extract on Recognition Memory

#### 3.10.1. Exploration Time in the NOR Test

**Figure 2:**
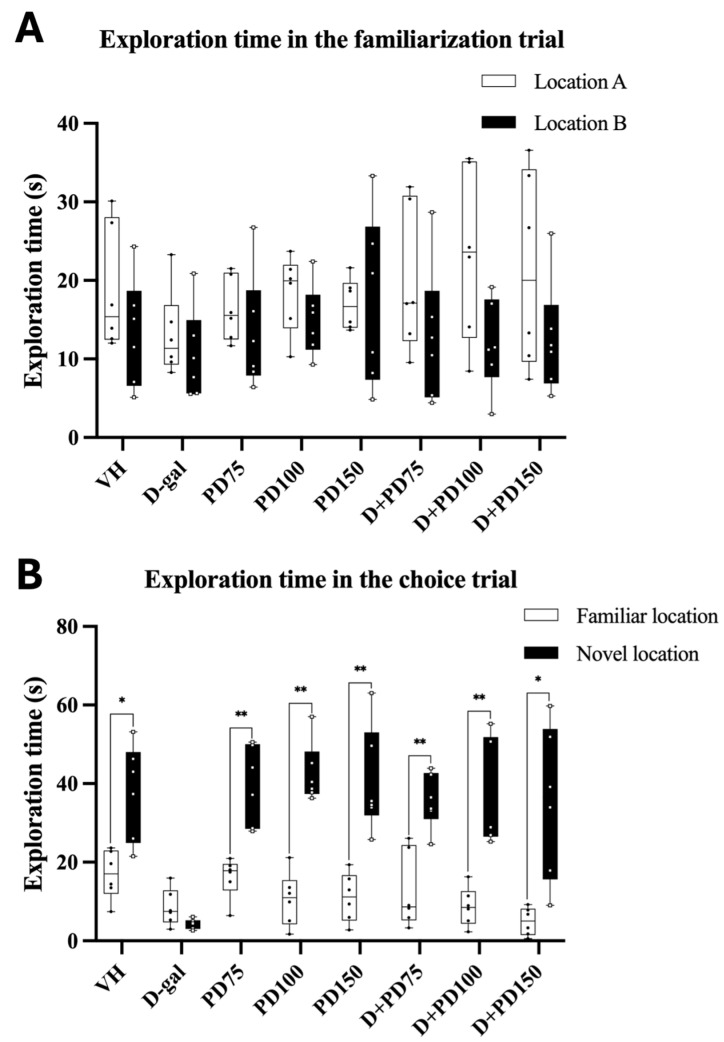
Exploration times of rats in the novel object recognition (NOR) test. (**A**) Exploration time during the familiarization trial (Location A vs. Location B). (**B**) Exploration time during the choice trial (Familiar vs. Novel location). Data are presented as box plots with individual data points (n = 12/group). The horizontal line indicates the median; boxes represent the interquartile range (IQR); whiskers show minimum–maximum values. Paired t-tests were used to compare exploration times between familiar and novel objects in the choice trial. Statistical significance is indicated as * *p* < 0.05 and ** *p* < 0.01. Effect sizes (Cohen’s d) with 95% confidence intervals are provided in Supplementary Table S2. Abbreviations: VH, vehicle; D-gal, D-galactose; PD, *Prunus domestica* L.

In the familiarization trial, there was no significant difference in the exploration time of objects A and B across all groups (*p >* 0.05, paired Student’s *t*-test, Figure 2A), indicating an equal preference for both objects. During the choice trial, rats in the vehicle, PD (75, 100, and 150 mg/kg), and D-gal + PD (75, 100, and 150 mg/kg) groups spent significantly more time exploring the novel object than the familiar one (*p <* 0.05, *p <* 0.01; Figure 2B), reflecting preserved recognition memory. In contrast, the D-gal-treated group showed no significant difference in exploration time between the two objects (*p >* 0.05; Table S2), suggesting impaired object recognition memory. These findings suggest that co-administration of PD with D-gal attenuated D-gal-induced deficits in object recognition.

#### 3.10.2. Preference Index (PI) in the NOR Test

**Figure 3:**
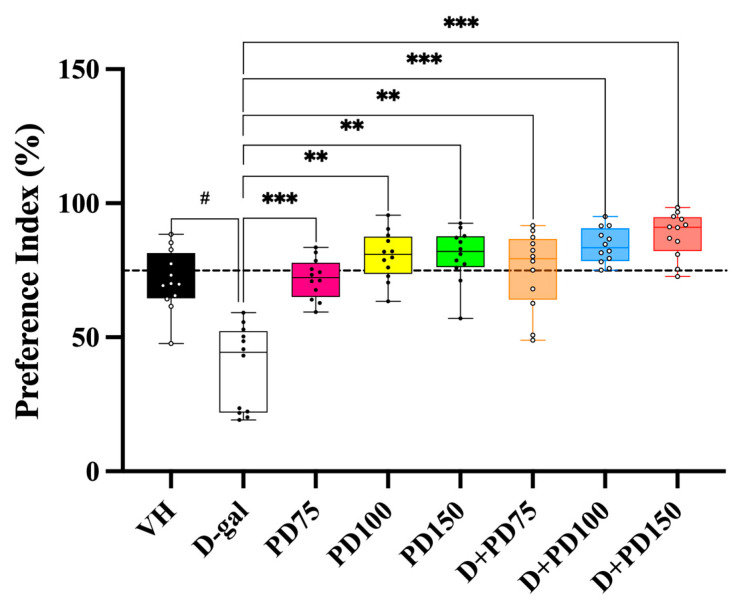
Preference index (PI) in the novel object recognition (NOR) test. Data are presented as box plots with individual data points (n = 12/group). The horizontal dashed line represents the 50% chance level. The line within each box indicates the median; boxes represent the interquartile range (IQR); whiskers show minimum–maximum values. One-sample *t*-tests were used to compare each group’s PI value against the 50% chance level. Statistical significance is indicated as ^#^ *p* < 0.05 compared with the vehicle group and ** *p* < 0.01, *** *p* < 0.001 compared with the D-gal group. Effect sizes (Cohen’s d) with 95% confidence intervals are provided in Supplementary Table S3. Abbreviations: VH, vehicle; D-gal, D-galactose; PD, *Prunus domestica* L.

The preference value reflects the natural tendency of rats to spend more time exploring new objects than familiar ones. The Preference Index (PI) was calculated by dividing the time spent exploring the novel object by the total exploration time spent exploring both objects and expressing this as a percentage. The PI is adjusted based on the time spent exploring the novel object, represented as a percentage relative to a 50% chance of equal preference. The PI values for the vehicle and PD-treated groups were significantly above the 50% chance level (*p* < 0.05, *p* < 0.01, *p* < 0.001, Figure 3), indicating a strong preference for the novel object and intact recognition memory. Similarly, rats receiving co-administration of PD and D-gal exhibited significantly higher PI values than the 50% chance level (*p* < 0.01, *p* < 0.001), suggesting that PD alleviated the memory impairment induced by D-gal. In contrast, the D-gal-treated group exhibited a PI below the 50% threshold (*p* > 0.05), indicating impaired recognition memory. These findings support a protective effect of PD against D-gal-induced cognitive deficits.

### 3.11. Effects of D-Gal and PD Crude Extract on Cell Proliferation in the Subgranular Zone (SGZ)

**Figure 4:**
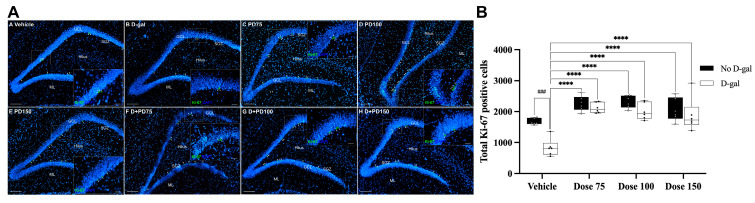
Ki-67–positive cells in the hippocampal dentate gyrus (DG). (**A**) Immunofluorescence images show Ki-67–positive nuclei (green) in the subgranular zone (SGZ) of the DG; nuclei are counterstained with DAPI (blue). White arrowheads indicate Ki-67–positive cells. Insets show higher magnification. Scale bar = 50 µm (40× objective; total magnification 400×). (**B**) Quantification of Ki-67–positive cells across treatment groups. Data are presented as box plots with individual data points (n = 6/group). The line within each box indicates the median; boxes represent the interquartile range (IQR); whiskers show minimum–maximum values. Two-way ANOVA (factors: D-gal presence/absence and PD dose) revealed significant main effects of D-gal (F(1, 40) = 17.76, η^2^_p_ = 0.307, *p =* 0.0001) and PD dose (F(3, 40) = 25.69, η^2^_p_ = 0.658, *p <* 0.0001), as well as a significant D-gal × PD dose interaction (F(3, 40) = 3.469, η^2^_p_ = 0.206, *p =* 0.0249). Bonferroni’s post hoc test: ^###^ *p* < 0.001 vs. Vehicle; **** p < 0.0001 vs. D-gal. Black boxes represent groups without D-gal administration, while white boxes indicate groups co-treated with D-galactose. Abbreviations: SGZ, subgranular zone; GCL, granule cell layer; ML, molecular layer; VH, vehicle; D-gal, D-galactose; PD, *Prunus domestica* L.

The Ki-67 protein is expressed throughout the active mitotic process of cells, indicating cell proliferation. We performed immunofluorescent labeling for the Ki-67 marker to measure proliferating cells in the SGZ of the hippocampal dentate gyrus (DG) (Figure 4A). Two-way ANOVA revealed a significant main effect of D-gal treatment (F(1, 40) = 17.76, *p =* 0.0001), a significant main effect of PD extract dose (F(3, 40) = 25.69, *p <* 0.0001), and a significant interaction between D-gal and PD extract dose (F(3, 40) = 3.469, *p =* 0.0249), indicating that the effect of the PD extract varied depending on D-gal exposure. D-gal administration significantly reduced the number of Ki-67 positive cells compared to the vehicle group (49.9%, *p =* 0.0006; Bonferroni’s post hoc test), indicating impaired cellular proliferation. Treatment with PD extract at 75, 100, and 150 mg/kg significantly increased the number of Ki-67 positive cells in D-gal-treated rats (153%, 141%, and 125%, respectively; all *p <* 0.0001; Figure 4B). These findings suggest that PD extract restores hippocampal cell proliferation under conditions of D-gal treatment.

### 3.12. Effect of D-Gal and PD Crude Extract on Superoxide Dismutase (SOD) Activity

**Figure 5:**
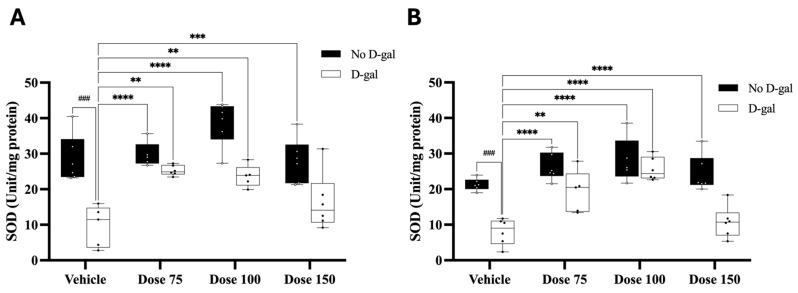
Quantification of SOD activity in the hippocampus and prefrontal cortex across experimental groups. (**A**) SOD activity in the hippocampus. (**B**) SOD activity in the prefrontal cortex. Data are presented as box plots with individual data points (n = 6/group). The line within each box indicates the median; boxes represent the interquartile range (IQR); whiskers show minimum–maximum values. Data were analyzed using two-way ANOVA (factors: D-gal presence/absence and PD dose), followed by Bonferroni’s post hoc test. Statistical significance: *^###^ p* < 0.001 vs. Vehicle; ** *p* < 0.01, *** *p* < 0.001, **** *p* < 0.0001 vs. D-gal. Black boxes represent groups without D-gal administration, while white boxes indicate groups co-treated with D-galactose. Full ANOVA statistics (F-values, *p*-values, and effect sizes η^2^_p_) are provided in Supplementary Table S4. Abbreviations: D-gal, D-galactose; PD, *Prunus domestica* L.

Two-way ANOVA revealed a significant interaction between D-gal treatment and PD extract dose on SOD activity in both the hippocampus (F(3, 37) = 2.869, *p =* 0.0495) and the prefrontal cortex (F(3, 38) = 4.174, *p =* 0.0119). Significant main effects of dose and D-gal treatment were also observed in the hippocampus (F(3, 37) = 8.898, *p =* 0.0001; F(1, 37) = 49.21, *p <* 0.0001) and in the prefrontal cortex (F(3, 38) = 17.26, *p <* 0.0001; F(1, 38) = 48.06, *p <* 0.0001). Post hoc analysis showed that D-gal significantly decreased SOD activity compared to the vehicle group in both the hippocampus (*p =* 0.0002) and the prefrontal cortex (*p =* 0.0002). PD treatment at 75 and 100 mg/kg significantly increased SOD activity in D-gal-treated rats in both the hippocampus and prefrontal cortex (*p <* 0.05, Figure 5A,B), while the 150 mg/kg dose did not produce a statistically significant effect (*p >* 0.9999). These results suggest that D-gal impairs antioxidant enzyme activity in both the hippocampus and prefrontal cortex, and that PD extract—particularly at moderate doses—can restore SOD activity depending on the presence or absence of oxidative stress.

## 4. Discussion

Most plants produce biologically active compounds, commonly referred to as phytochemical ingredients. Among these, phenolic compounds, particularly flavonoids, exhibit significant therapeutic benefits [38,39]. Our objective was to identify phytochemicals in the PD extract and assess their biological activities. Phenolic compounds and their derivatives possess potent antioxidant properties, largely attributed to the presence of molecular hydroxyl, carboxylic groups, and conjugated ring structures. These phenolic phytochemicals can donate hydrogen atoms to reactive oxygen species (ROS) and neutralize other free radicals, playing roles in multiple biological processes [9,40]. Given the antioxidant activities of phenolic compounds, it is well established that polyphenolic compounds present in fruits and vegetables potentially mitigate the onset or progression of various neurodegenerative diseases when consumed regularly [41,42]. In this study, LC–MS QTOF analysis was used to characterize the phytochemical constituents of the PD extract. The results indicated a diverse chemical profile consisting of phenolic and lipid-related metabolites in both positive and negative ionization modes [43]. The main compounds identified with high peak intensity and reliable spectral matches, included catechin, oleacein, mangostinone, garcinone A, myrsinone, and several fatty acids, specifically palmitic, hexadecanoic, and lithocholic acids. Catechin, a phenolic compound previously reported in *Prunus domestica* and related *Prunus species* [43,44,45], supports a key observation: the predominance of phenolic antioxidants within the extract. In addition, oleacein, mangostinone, garcinone A, and myrsinone are known bioactive metabolites in other plants, but their presence in the PD extract has not been described before. This new evidence expands the current phytochemical information for the species and suggests that these newly identified secoiridoid and xanthone derivatives may contribute to the extract’s antioxidant and neuroprotective potential [46,47,48].

Following phytochemical profiling, total phenolic content (TPC) was quantified using the Folin–Ciocalteu assay. In this study, we quantified the phenolic content in PD crude extract using the Folin–Ciocalteu assay. The extract showed a relatively high phenolic concentration, which aligns with its ability to reduce phosphomolybdic acid and form a blue molybdenum complex, thereby confirming the presence of antioxidant phenolics. This reinforces prior evidence that phenolic compounds are key contributors to antioxidant activity [49,50]. Among these phenolics, tannins were particularly abundant in the PD extract. The elevated tannin content is of special interest because tannins, which are high–molecular weight polyphenols, contain multiple hydroxyl groups that are efficient electron donors. This structural feature is consistent with the strong reducing activity observed in the FRAP assay, in which the extract showed the highest ferric-reducing capacity. Thus, the high tannin content likely underpins the enhanced FRAP values [40,51]. In contrast, the extract displayed only moderate radical-scavenging ability in the DPPH assay. This discrepancy can be explained by the steric limitations of bulky polyphenols such as tannins, which are less effective at accessing the radical site of DPPH compared to smaller phenolics or flavonoids. Such differences have also been reported in other polyphenol-rich extracts, reflecting the distinct mechanisms captured by each assay [52,53,54,55,56]. Specifically, DPPH reflects radical scavenging, whereas FRAP emphasizes electron transfer potential. Accordingly, the PD extract demonstrates moderate hydrogen-donating activity in DPPH but strong reducing power in FRAP, suggesting that its antioxidant effects are expressed more effectively through redox-based mechanisms rather than direct free radical quenching. Flavonoids, though present at lower levels than tannins, further contribute to antioxidant defense by neutralizing free radicals, inducing antioxidant enzymes, and acting as metal-chelating agents [57]. These functions are well documented in experimental and clinical studies, supporting their role in the moderate DPPH activity observed here [58,59,60]. Likewise, the detected anthocyanins are likely to enhance the extract’s redox balance, as these pigments are known to stabilize oxidative processes and are abundant in fruits such as plums [9,60]. Taken together, these findings tentatively suggest that the antioxidant potential of the PD extract is mediated primarily through redox-based electron transfer mechanisms rather than direct radical scavenging. The integration of LC–MS QTOF profiling with colorimetric assays provides a comprehensive understanding of its phytochemical diversity and biological activity. The identification of new secoiridoid and xanthone derivatives alongside phenolic compounds highlights the PD extract as a promising natural source of antioxidant and neuroprotective agents.

Memory impairment is more prevalent among the elderly, and it is posited that age-related memory decline correlates with decreased neurogenesis in the adult hippocampus [60,61]. Recent studies indicate that chronic administration of D-gal accelerates aging and leads to cognitive decline in rodents. The co-administration of D-gal and aluminum chloride in rats is ideal for mimicking Alzheimer’s disease-like cognitive impairments. D-gal is a senescence agent, while aluminum is a well-known neurotoxin associated with the pathogenesis of Alzheimer’s disease [62]. Consequently, D-gal has gained acceptance for research focused on brain aging using models that mimic the natural aging process. D-gal has been found to reduce neurogenesis in the rat hippocampus, resulting in cognitive deficits as measured by the object recognition test [63,64,65]. Object recognition memory, a subset of the non-spatial domain of declarative memory, refers to the capability to recognize and remember prior objects and experiences [66]. The novel object recognition (NOR) test serves as a behavioral measure of non-spatial memory. This assessment leverages a rodent’s inherent exploratory behavior absent external positive or negative reinforcement. Rodents will instinctively spend more time exploring novel over familiar stimuli. Given this predisposition, the NOR test is a robust method for determining neurobiological underpinnings supporting hippocampus-associated memory formation [67]. Although other paradigms such as the Y-maze and Morris water maze are widely employed to assess hippocampus-dependent spatial learning and memory, the present study specifically focused on non-spatial recognition memory associated with hippocampal–perirhinal circuitry. Accordingly, the NOR test was deemed more appropriate for evaluating recognition memory deficits in D-gal–treated rats, as it provides a sensitive yet low-stress measure of object recognition that is minimally influenced by motivational or swimming-related factors [31,32]. Numerous studies suggest that the perirhinal cortex underlies object recognition memory, while the hippocampus plays a pivotal role, given its intimate association with the perirhinal cortex [68,69,70]. In various adult rat models, brain aging induced by sustained administration of D-gal (50 mg/kg, i.p.) led to deficits in recognition memory. This decline is attributed to reductions in cell proliferation, survival, and the presence of immature neurons during hippocampal neurogenesis, highlighting D-gal’s role in these recognition memory impairments [31,32]. Our observations regarding the detrimental effects of D-gal align with prior research findings of a correlation between D-gal-induced hippocampal injury and non-spatial memory impairment. The preference index for rats treated with D-gal alone was below 50%, signaling a deficit in recognition memory. Plums (*Prunus domestica* L.) are a rich source of antioxidants, encompassing phenolic acids, anthocyanins, and other flavonoids [71]. A growing body of research suggests that *Prunus domestica* L. (PD) contributes significantly to dietary antioxidant intake. Notably, PD boasts a rich flavonoid profile. These flavonoids are theorized to bolster memory performance by modulating the hippocampal dentate gyrus, which in turn affects various neuronal signaling pathways. Previous studies have indicated that polyphenols and anthocyanins activate the Nrf2/HO-1 pathway, thereby enhancing endogenous antioxidant defenses and reducing ROS accumulation [10]. Furthermore, flavonoid-rich interventions stimulate BDNF/TrkB signaling, which supports neuronal survival and synaptic plasticity, and increase CREB phosphorylation to facilitate memory consolidation [72,73,74]. Because PD is rich in polyphenols and anthocyanins, these mechanisms provide a molecular rationale for the neuroprotective and neurogenic potential. Thus, the neuroprotective effects of PD might counteract learning and recognition memory deficits by enhancing hippocampal neurogenesis via the antioxidant capacity of flavonoids [75,76,77]. Our results showed that PD crude extract, at all concentrations and in combination with D-gal groups, augmented recognition memory. This observation is consistent with prior findings that PD extracts enhance learning and memory efficacy in both healthy mice and aged rats in behavioral assessments [8,78]. Recognition memory, as assessed by the NOR test, relies heavily on the functioning of both the hippocampus and the prefrontal cortex. The hippocampus is mainly responsible for memory encoding and detecting novelty, while the prefrontal cortex plays a role in attention modulation and decision-making during object exploration [67]. Administration of D-gal at clinically relevant doses has been found to induce apoptotic neurodegeneration in several brain regions, especially in the hippocampus and prefrontal cortex. Cumulative evidence indicates that the neurotoxic effects of D-gal are largely mediated through oxidative stress and the downregulation of endogenous antioxidant defense mechanisms, including enzymes like superoxide dismutase (SOD) [79]. In line with previous reports, the present study demonstrated that D-gal-induced oxidative stress significantly reduced SOD activity in both the hippocampus and prefrontal cortex, which may partly account for the observed deficits in recognition memory. Notably, co-administration of PD extract at doses of 75 and 100 mg/kg effectively restored SOD activity levels, highlighting its potential neuroprotective effects through enhancement of antioxidant defenses. However, the highest dose (150 mg/kg) did not confer additional benefits in D-gal-treated rats. The significant interaction between D-gal and PD dose does suggest that the antioxidant effects of PD are modulated by the tissue’s oxidative state. Although PD extract exhibited significant neuroprotective and antioxidant effects at all tested doses, the relationship was not strictly dose-dependent. The 75 and 100 mg/kg doses produced consistent improvements in SOD activity, whereas a higher dose (150 mg/kg) did not further enhance the outcomes. This pattern suggests a nonlinear or plateaued dose–response relationship rather than a loss of efficacy. Similar phenomena have been reported for other polyphenol-rich extracts, where moderate doses are sufficient to activate antioxidant pathways, while higher doses provide no additional benefit due to limited bioavailability and metabolic saturation [80,81]. Moreover, excessive polyphenol exposure has been reported to exert mild pro-oxidant or antagonistic effects that can transiently suppress endogenous antioxidant enzymes [82,83]. The in vitro cytotoxicity data in this study also revealed that PD extract was non-toxic up to 125 µg/mL but showed mild cytotoxicity at concentrations above 250 µg/mL, supporting the possibility that very high doses could impose a metabolic burden. Although no in vivo toxicity or behavioral abnormalities were observed, chronic administration of supraphysiological doses may challenge hepatic conjugation and microbial metabolism of polyphenols. Hence, 75–100 mg/kg likely represents an optimal physiological window that achieves maximal neuroprotection and antioxidant benefit while minimizing metabolic stress. Furthermore, at these effective doses, the restoration of antioxidant activity by PD is consistent with activation of the Nrf2/HO-1 pathway, which regulates endogenous antioxidant enzymes and contributes to neuronal protection. In the dentate gyrus, D-gal reduced Ki-67 proliferating cells, whereas coadministration with PD increased their numbers, indicating enhanced neurogenesis. These findings align with reports that flavonoids, which are also found in PD, stimulate BDNF/TrkB-CREB signaling and support neuronal survival, differentiation, and hippocampal plasticity. Although BDNF and CREB were not directly measured, the observed improvements in SOD activity and cell proliferation strongly imply that PD promotes hippocampal neurogenesis through the involvement of these pathways. Consistent with previous studies, our results showed that D-gal exposure significantly reduced cell proliferation in the hippocampal dentate gyrus, as indicated by decreased Ki-67 immunoreactivity [31,32]. Ki-67 is a well-established endogenous marker of proliferating cells, expressed throughout all active phases of the cell cycle [84]. Chronic administration of D-gal has been reported to inhibit hippocampal neurogenesis by inducing oxidative stress and lipid peroxidation, which in turn trigger apoptotic pathways and reduce the survival of newly generated neurons in the SGZ [85]. Since the hippocampus is particularly vulnerable to age-related oxidative stress, D-gal has been widely used to model aging-related cognitive and cellular decline. Importantly, co-administration of PD extract at doses of 75, 100, and 150 mg/kg markedly increased the number of Ki-67 positive cells in D-gal-treated rats, suggesting a restorative effect on neurogenic activity. The significant interaction between D-gal and PD dose indicates that PD’s pro-proliferative effects are enhanced under neurodegenerative conditions, possibly through upregulation of antioxidant defenses or neurotrophic signaling pathways. These findings suggest that PD may improve hippocampal neurogenesis and mitigate D-gal-induced impairment, which is consistent with behavioral outcomes observed in related studies [31,32,86,87]. Brain-derived neurotrophic factor (BDNF) is essential for neuronal development [13,88]. Studies show that D-gal-treated rats exhibit suppressed BDNF expressions [79]. However, while specific evidence for PD enhancing neurotrophins is lacking, several related polyphenolic compounds (including quercetin, curcumin, resveratrol, and hydroxytyrosol) have been demonstrated to activate BDNF signaling [89]. Therefore, a potential mechanism by which PD improves hippocampal neurogenesis in animals receiving D-gal treatment could be the modulation of the BDNF signaling pathway. The present findings demonstrate that D-gal administration significantly impairs hippocampal neurogenesis and antioxidant defense mechanisms, as evidenced by a reduction in Ki67 positive cells and decreased SOD activity in both the hippocampus and prefrontal cortex. Notably, the significant interaction between D-gal treatment and PD dosage indicates that the neuroprotective and antioxidant effects of PD are both dose-dependent and context-specific, potentially reflecting a differential response under oxidative stress conditions. Previous research indicated that daily oral doses of Prunus fruit extracts for seven days can enhance learning and memory [14,78]. Recent studies have suggested that high consumption of plums, rich in phenolic compounds, may positively impact memory-related issues, reduce inflammation, and scavenge ROS. Ethanol-based PD extract therapy has been shown to alleviate oxidative stress in an Alzheimer’s model [8,14]. Together, these findings highlight the antioxidant properties of PD extract in mitigating D-gal-induced cellular damage and underscore its dual action as a neurogenic agent. A key translational consideration, however, is the limited bioavailability of PD’s constituent phytochemicals. Although PD is rich in chlorogenic acids, caffeic acid derivatives, anthocyanins, and flavonoids, their concentrations decline significantly during gastrointestinal digestion, particularly in the later intestinal stages [9]. Absorbed compounds undergo rapid phase II metabolism, whereas unabsorbed fractions are converted by gut microbiota into smaller catabolites that may still retain biological activity [90]. Pharmacokinetic studies confirm low plasma levels and short half-lives for the parent compounds, suggesting that conjugated metabolites and microbial catabolites are likely the primary mediators of PD’s neuroprotective effects [91]. Despite these bioavailability limitations, clinical evidence consistently demonstrates the beneficial effects of plum products on cognition. Intervention studies utilizing plum juice and high-anthocyanin varieties have shown a range of outcomes, including improvements in plasma antioxidant status, increased urinary metabolites, reductions in oxidative stress biomarkers, and enhancements in certain cognitive outcomes [92,93]. Dose translation from rodent studies suggests that an effective intake may be approximately 1–2 g/day of flavonoids, equivalent to consuming several plums or about 400 mL of juice [73,93]. Human interventions also indicate good tolerability; however, excessive intake of dried plums may cause mild gastrointestinal discomfort due to their sorbitol and fiber content. Based on the dosage conversion method by Nair and Jacob, PD at doses 75 and 100 mg/kg was effective in improving memory, hippocampal neurogenesis, and SOD activity in D-gal-treated rats. The 100 mg/kg was therefore selected to calculate the Human Equivalent Dose, considering conversion factors and the No Observed Adverse Effect Levels [94]. Collectively, these data results support the therapeutic potential of PD in counteracting D-gal-induced neurodegeneration, likely through modulation of redox balance and stimulation of endogenous cell proliferation pathways.

## 5. Conclusions

This study demonstrated that, while D-gal adversely affects recognition memory in treated rats, concurrent administration with PD appears to mitigate these effects. This is potentially due to the antioxidant properties of PD. Flavonoids may exert influence over various neurological functions, notably augmenting cognitive capabilities. Importantly, our data reveal the presence of flavonoid compounds within the PD crude extract. We propose that the neuroprotective attributes of PD may counteract the deficits in recognition memory, potentially driven by the specific concentrations of flavonoid compounds present. These results highlight the necessity for additional research into the cellular mechanisms that underpin the influence of PD on the signaling pathways within hippocampal neurogenesis tied to cognitive function.

## Figures and Tables

**Table 1:** Cytotoxic activity and the IC50 value of *Prunus domestica* L. (PD) crude extracts on mouse macrophage-like cell line RAW264.7.

No	Sample Name	Concentration (μg/mL)	Cell Viability (%)	IC50 (μg/mL)
1	Control	0	100.00 ± 1.03	
2	PD crude extract	3.91	101.19 ± 4.16	>500.00
		7.81	100.84 ± 1.22	
		15.63	99.46 ± 0.91	
		31.25	94.41 ± 3.67	
		62.50	91.54 ± 1.32	
		125.00	88.87 ± 2.04	
		250.00	74.82 ± 0.60	
		500.00	72.07 ± 3.38	

## Data Availability

All data supporting the findings of this study are available within the article and can be accessed without restrictions.

## Supplementary Materials

The following supporting information can be downloaded at: https://www.mdpi.com/article/10.3390/nu17233804/s1, Figure S1. UHPLC-QTOF-MS chromatograms of *Prunus domestica* L. extract in (A) positive and (B) negative ionization modes. Table S1: Chemical profile of *Prunus domestica* L. extract identified by UHPLC-QTOF-MS in positive and negative ionization modes. Table S2: Exploration time of rats in the novel object recognition (NOR) test during familiarization and choice trials, with *p*-values and effect sizes; Table S3: Preference index (PI) in the NOR test, with statistical analysis; Table S4: Two-way ANOVA results for antioxidant enzyme activity; and Supplementary File S1 describing sample size and power calculations.
