# Supplementary material for: Oral Supplementation with Prunus domestica L. Extract Restores Recognition Memory Impairment Caused by D-Galactose in Rats
Source: Nutrients. 2025 Dec 4;17(23):3804. doi: 10.3390/nu17233804 (PMC12693760; doi:10.3390/nu17233804)
Supplement: Supplementary file 1 [file nutrients-17-03804-s001.zip › nutrients-4005104-supplementary.pdf]

**Figure S1. UHPLC-QTOF-MS chromatograms of *Prunus domestica* L. extract in (A) positive and (B) negative ionization modes.**

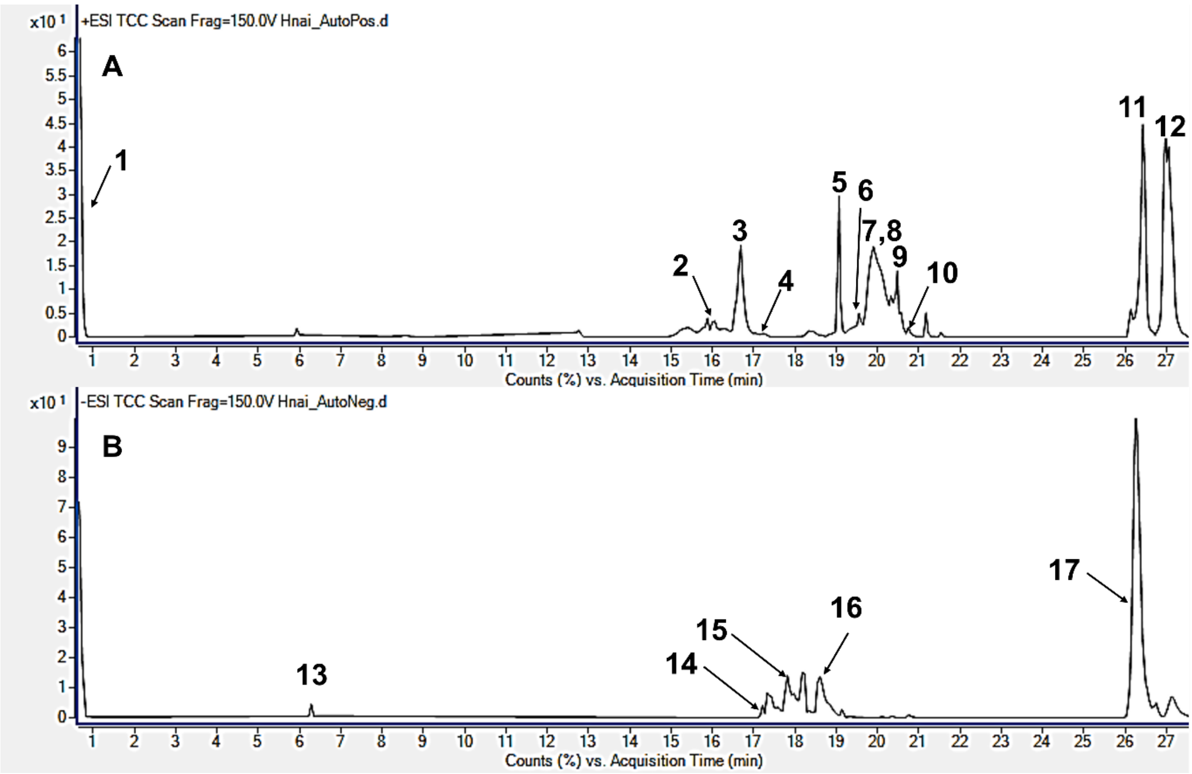

**Table S1. Chemical profile of *Prunus domestica* L. extract identified by UHPLC-QTOF-MS in positive and negative ionization modes.**

| No. | $t_R$ (min) | mass | *m/z* | Chemical formular | Compounds name | Class |
|---|---|---|---|---|---|---|
| Positive mode | | | | | | |
| **1** | 0.646 | 342.116 | 360.1498 | C12 H22 O11 | Sucrose | Sugar |
| **2** | 16.01 | 320.1269 | 343.1163 | C17 H20 O6 | Oleacein | Phenolic |
| **3** | 16.68 | 270.2558 | 288.2896 | C17 H34 O2 | Hexadecanoic acid | saturated fatty acid |
| **4** | 17.23 | 210.2344 | 228.2683 | C15 H30 | (*E*)-7-Pentadecene | long-chain unsaturated fatty acid |
| **5** | 19.06 | 204.0783 | 205.0857 | C12 H12 O3 | Anofinic acid | benzopyran |
| **6** | 19.55 | 286.2146 | 309.2038 | C16 H30 O4 | Adipic acid | dicarboxylic acid |
| **7** | 19.86 | 256.2401 | 274.274 | C16 H32 O2 | Palmitic acid | saturated fatty acid |
| **8** | 20.27 | 289.2618 | 290.2688 | C16 H35 N O3 | Lauric acid | saturated fatty acid |
| **9** | 20.48 | 278.2607 | 296.2946 | C19 H34 O | 2-Pentadecylfuran | pentadecyl furan |
| **10** | 20.74 | 376.2978 | 394.3316 | C24 H40 O3 | Lithocholic acid | Triterpene |
| **11** | 26.43 | 270.2558 | 288.2896 | C17 H34 O2 | Ethyl pentadecanoate | long-chain fatty ester |
| **12** | 27.10 | 226.2294 | 244.2632 | C15 H30 O | Pentadecanal | long-chain fatty aldehyde |
| Negative mode | | | | | | |
| **13** | 6.280 | 290.0791 | 289.0718 | C15 H14 O6 | Catechin | Phenolic |
| **14** | 17.21 | 294.1829 | 293.1756 | C17 H26 O4 | Myrsinone | 1,4-benzoquinone |
| **15** | 17.83 | 380.1628 | 379.1559 | C23 H24 O5 | Mangostinone | Xanthone |
| **16** | 18.54 | 234.1617 | 233.1544 | C15 H22 O2 | Dihydro isoalantolactone | Ssquiterpene lactone |
| **17** | 26.28 | 380.1626 | 379.1553 | C23 H24 O5 | Garcinone A | Xanthone |

**Table S2: Exploration time of rats in the novel object recognition (NOR) test during familiarization and choice trials, with p-values and effect sizes (Cohen's d, 95% CI).**

| Group | Exploration time (s) | | | | p-value (NO vs FO) | Cohen's d [95% CI] | Interpretation |
|---|---|---|---|---|---|---|---|
| | Familiarization trial | | Choice trial | | | | |
| | Location A | Location B | Familiar object (FO) | Novel object (NO) | | | |
| Vehicle | 18.8 ± 3.2 | 13.3 ± 2.9 | 16.9 ± 2.5 | 37.9 ± 4.9 * | 0.008 | 1.82 [0.85, 2.67] | Very large |
| D-gal | 13.4 ± 2.2 | 13.5 ± 4.0 | 9.0 ± 2.0 | 4.3 ± 0.5 | 0.121 (n.s.) | –1.09 [–1.90, –0.28] | Large (negative) |
| PD 75 mg | 17.3 ± 1.7 | 14.1 ± 2.7 | 16.2 ± 2.1 | 39.7 ± 4.1 ** | 0.004 | 2.11 [1.12, 2.85] | Very large |
| PD 100 mg | 17.4 ± 2.1 | 14.9 ± 1.9 | 10.6 ± 2.8 | 41.6 ± 4.4 ** | 0.002 | 2.49 [1.35, 3.27] | Very large |
| PD 150 mg | 17.0 ± 1.3 | 17.1 ± 4.5 | 11.0 ± 2.5 | 40.4 ± 5.5 ** | 0.003 | 2.36 [1.21, 3.09] | Very large |
| D-gal + PD 75 mg | 19.9 ± 3.8 | 12.8 ± 3.6 | 12.7 ± 3.9 | 35.7 ± 2.9 ** | 0.006 | 1.67 [0.76, 2.45] | Large |
| D-gal + PD 100 mg | 23.4 ± 4.4 | 11.9 ± 2.4 | 8.7 ± 2.0 | 39.6 ± 5.7 ** | 0.003 | 2.22 [1.18, 3.00] | Very large |
| D-gal + PD 150 mg | 21.3 ± 5.1 | 13.9 ± 6.7 | 4.9 ± 1.4 | 40.3 ± 11.7 * | 0.012 | 1.56 [0.65, 2.33] | Large |

*$p$□0.05, and **$p$□0.01 significant difference compared to familiar location

FO: Familiar object; NO: Novel object; NOR: Novel object recognition; D-gal: D-galactose; PD: *Prunus domestica* L.

**Table S3: Preference index (PI) in the novel object recognition (NOR) test, with p-values and effect sizes (Cohen's d, 95% CI)**

| Group | PI (Mean ± SEM) | p-value vs. 50% | Cohen's d [95% CI] | Interpretation |
|---|---|---|---|---|
| Vehicle | 68.38 ± 5.24* | 0.004 | 1.53 [0.63, 2.28] | Large |
| D-gal | 36.09 ± 6.97 | 0.082 (n.s.) | –0.58 [–1.28, 0.10] | Medium (negative) |
| PD 75 mg | 71.28 ± 3.04*** | 0.001 | 2.11 [1.12, 2.87] | Very large |
| PD 100 mg | 80.68 ± 4.47** | <0.001 | 2.73 [1.58, 3.46] | Very large |
| PPD 150 mg | 78.80 ± 4.94** | <0.001 | 2.47 [1.35, 3.19] | Very large |
| D-gal + PD 75 mg | 75.47 ± 6.31** | 0.002 | 1.99 [1.05, 2.73] | Large |
| D-gal + PD 100 mg | 82.30 ± 3.10*** | <0.001 | 3.00 [1.89, 3.72] | Very large |
| D-gal + PD 150 mg | 87.93 ± 3.76*** | <0.001 | 2.85 [1.73, 3.59] | Very large |

*p < 0.05, **p < 0.01, ***p < 0.001 vs. 50% chance level. VH: vehicle; D-gal: D-galactose; PD: *Prunus domestica* L.

**Table S4: Two-way ANOVA results for SOD activity in hippocampus and prefrontal cortex, with F-values, p-values, and effect sizes (partial $\eta^2$).**

| Brain region | Factor | df | F | p-value | $\eta^2_p$ |
|---|---|---|---|---|---|
| Hippocampus | D-gal (main) | 1,37 | 49.21 | <0.0001 | 0.571 |
| | Dose (main) | 3,37 | 8.898 | 0.0001 | 0.419 |
| | Interaction | 3,37 | 2.869 | 0.0495 | 0.189 |
| Prefrontal cortex | D-gal (main) | 1,38 | 48.06 | <0.0001 | 0.558 |
| | Dose (main) | 3,38 | 17.26 | <0.0001 | 0.577 |
| | Interaction | 3,38 | 4.174 | 0.0119 | 0.248 |

**Supplementary File S1: Sample Size and Power Calculation**

To determine the minimum number of animals required per group for adequate statistical power in comparing the mean values between the *Control* and *D-gal* groups using a **two-sample, two-sided t-test (**data from Zhao et al. (2019)**)**.

**Assumptions and Parameters**

| Parameter | Description | Symbol |
|---|---|---|
| Significance level (two-sided) | α | 0.01 |
| Corresponding critical value | $Z_{\alpha/2}$ | 2.58 |
| Desired power | 1−β | 0.80 |
| Corresponding Z value | $Z_{\beta}$ | 0.84 |
| Control group mean | — | 63.44 |
| D-gal group mean | — | 40.22 |
| Control group SD | $SD_1$ | 17.42 |
| D-gal group SD | $SD_2$ | 12.50 |

**Formula Used** For a two-sample, two-sided t-test (assuming equal variances):

$$N = \frac{(Z_{\alpha/2} + Z_{\beta})^2 \cdot 2\sigma^2}{\Delta^2}$$

where:
$\sigma$ = pooled standard deviation
$\Delta$ = expected difference between means

**Step 1. Calculate the Pooled Standard Deviation ($\sigma$)**

$$\sigma^2 = \frac{SD_1^2 + SD_2^2}{2}$$

$$= \frac{17.42^2 + 12.50^2}{2} = \frac{303.4564 + 156.25}{2} = \frac{459.7064}{2} = 229.8532$$

$$\sigma = \sqrt{229.8532} \approx 15.16$$

**Step 2. Calculate the Mean Difference ($\Delta$)**

$$\Delta = 63.44 - 40.22 = 23.22$$

**Step 3. Substitute into the Sample Size Formula**

$$(Z_{\alpha/2} + Z_{\beta}) = 2.58 + 0.84 = 3.42$$

$$(Z_{\alpha/2} + Z_{\beta})^2 = 3.42^2 = 11.6964$$

$$2\sigma^2 = 2 \times 229.8532 = 459.7064$$

$$\Delta^2 = 23.22^2 = 539.1684$$

$$N = \frac{11.6964 \times 459.7064}{539.1684} = \frac{5376.91}{539.17} \approx 9.97$$

$$\boxed{N = 10 \text{ animals per group (rounded up)}}$$

**Step 4. Calculate the Effect Size (Cohen's d)**

$$d = \frac{\Delta}{\sigma} = \frac{23.22}{15.16} \approx 1.53$$

This corresponds to a **large effect size** according to Cohen's convention.

**Summary**

| **Parameter** | **Symbol** | **Value** |
|---|---|---|
| Significance level (two-sided) | α | 0.01 |
| Power | 1−β | 0.80 |
| Pooled SD | σ | 15.16 |
| Mean difference | Δ | 23.22 |
| Effect size (Cohen's d) | d | 1.53 |

- Sample size was rounded **upward** to ensure adequate statistical power.
- An additional **10–20% of animals** were included to compensate for potential attrition or experimental loss.
- The calculation assumes **equal variance** and **normal distribution** between groups.
- If actual variances differ substantially, Welch's correction and a revised power analysis may be required.
